# Author Correction: The future of Southeast Asia’s forests

**DOI:** 10.1038/s41467-023-41060-9

**Published:** 2023-08-30

**Authors:** Ronald C. Estoque, Makoto Ooba, Valerio Avitabile, Yasuaki Hijioka, Rajarshi DasGupta, Takuya Togawa, Yuji Murayama

**Affiliations:** 1 National Institute for Environmental Studies, Tsukuba, Japan; 2 European Commission, Joint Research Centre (JRC), Ispra, Italy; 3 Institute for Global Environmental Strategies, Kanagawa, Japan; 4 University of Tsukuba, Tsukuba, Japan

Correction to: *Nature Communications* 10.1038/s41467-019-09646-4, Article published online 23 April 2019

The original version of this Article contained an error in the Data availability statement, which incorrectly read ‘The sources of all the data used are given in the Methods section. All the geospatial data associated with the results presented, as well as the R code used, are available from the first and corresponding author upon request.’ The correct version states ‘The sources of all the data used are given in the Methods section. The raster dataset for the simulated future forest cover losses and gains across the five baseline SSPs are available at 10.5281/zenodo.8162177’. This has been corrected in both the PDF and HTML versions of the Article.

