# Supplementary material for: The future of Southeast Asia’s forests
Source: Nat Commun. 2019 Apr 23;10:1829. doi: 10.1038/s41467-019-09646-4 (PMC6478739; doi:10.1038/s41467-019-09646-4)
Supplement: Supplementary file 1 — Supplementary Information [file 41467_2019_9646_MOESM1_ESM.pdf]

The Future of Southeast Asia's Forests

Estoque *et al.*

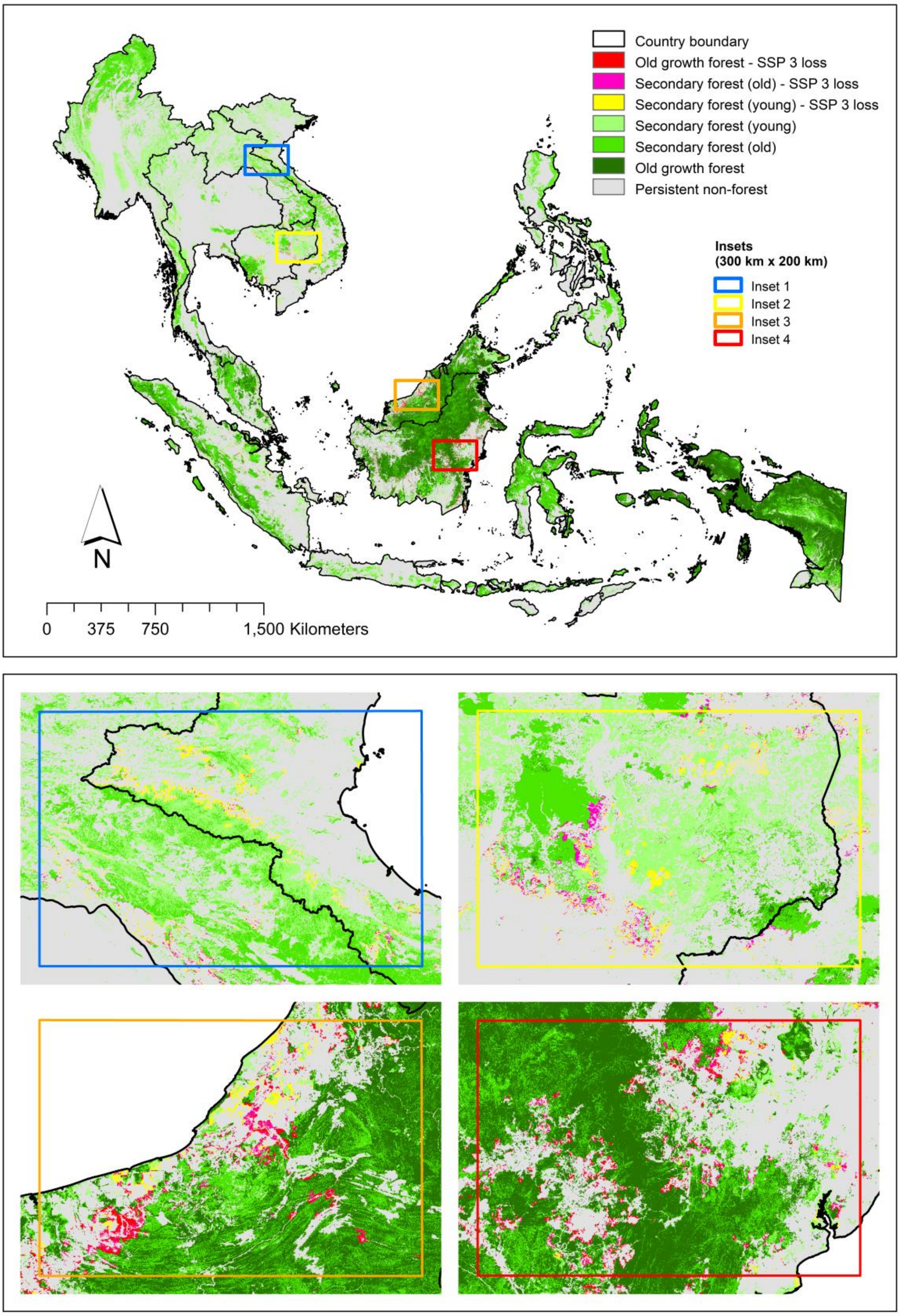

Supplementary Figure 1 Projected forest cover losses across forest classes in Southeast Asia under the worst-case scenario (SSP 3) (2015-2050). See Fig. 4 for corresponding statistics. The four insets show the spatially allocated projected forest cover changes in some parts of Laos and Vietnam (inset 1), Cambodia (inset 2), Malaysia (inset 3) and Indonesia (inset 4)

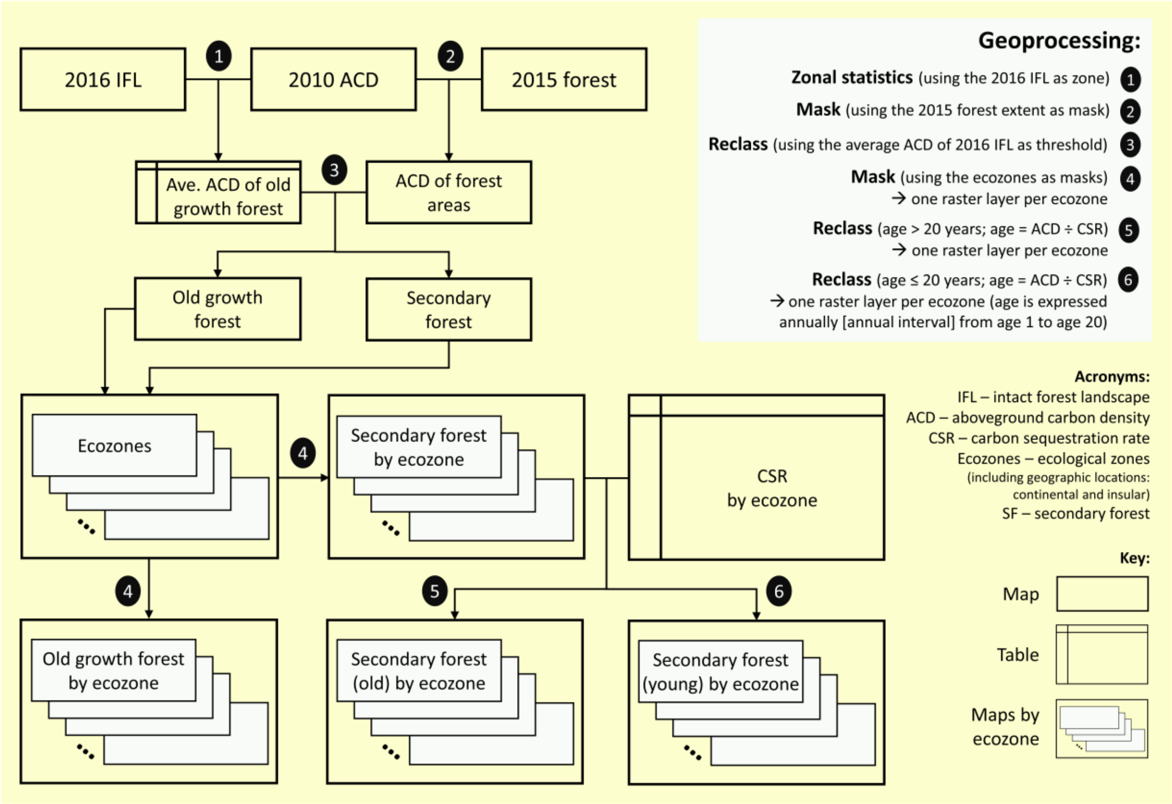

Supplementary Figure 2 Geospatial approach developed and used to reclassify the forest class. The approach considers intact forest landscapes (IFLs), aboveground carbon density (ACD), carbon sequestration rates (CSRs), ecological zones (ecozones) and geographic locations (continental and insular). The average ACD of the 2016 IFL was 121 Mg C ha^{-1}. All the geoprocessing steps were performed in ArcMap 10.5

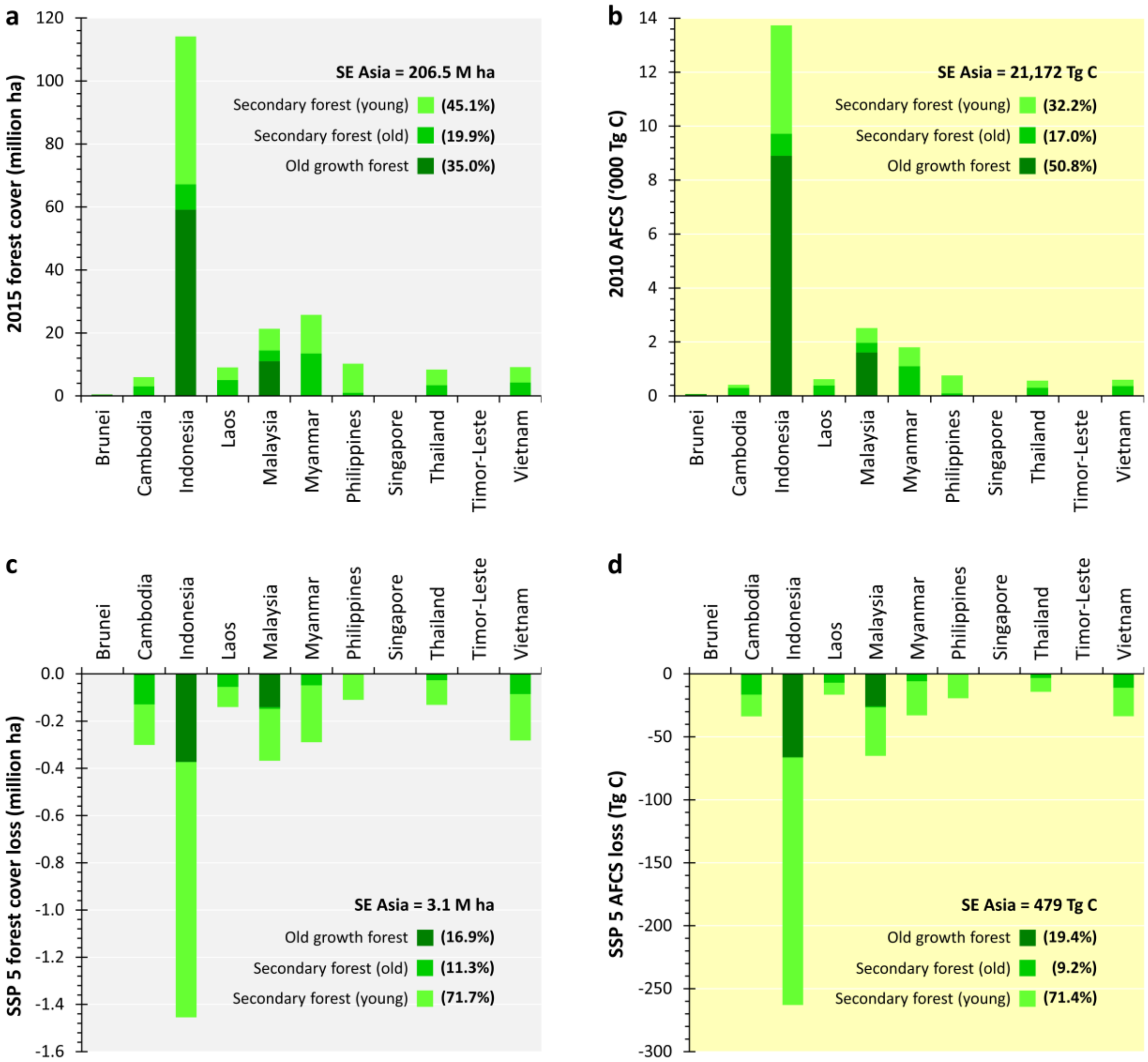

Supplementary Figure 3 Forest cover and AFCS in Southeast Asia, and their respective losses by 2050 across forest classes under SSP 5. Country-level distribution of forest cover and AFCS considering forest classes (**a**, **b**) and country-level distribution of projected forest cover and AFCS losses across forest classes (**c**, **d**)

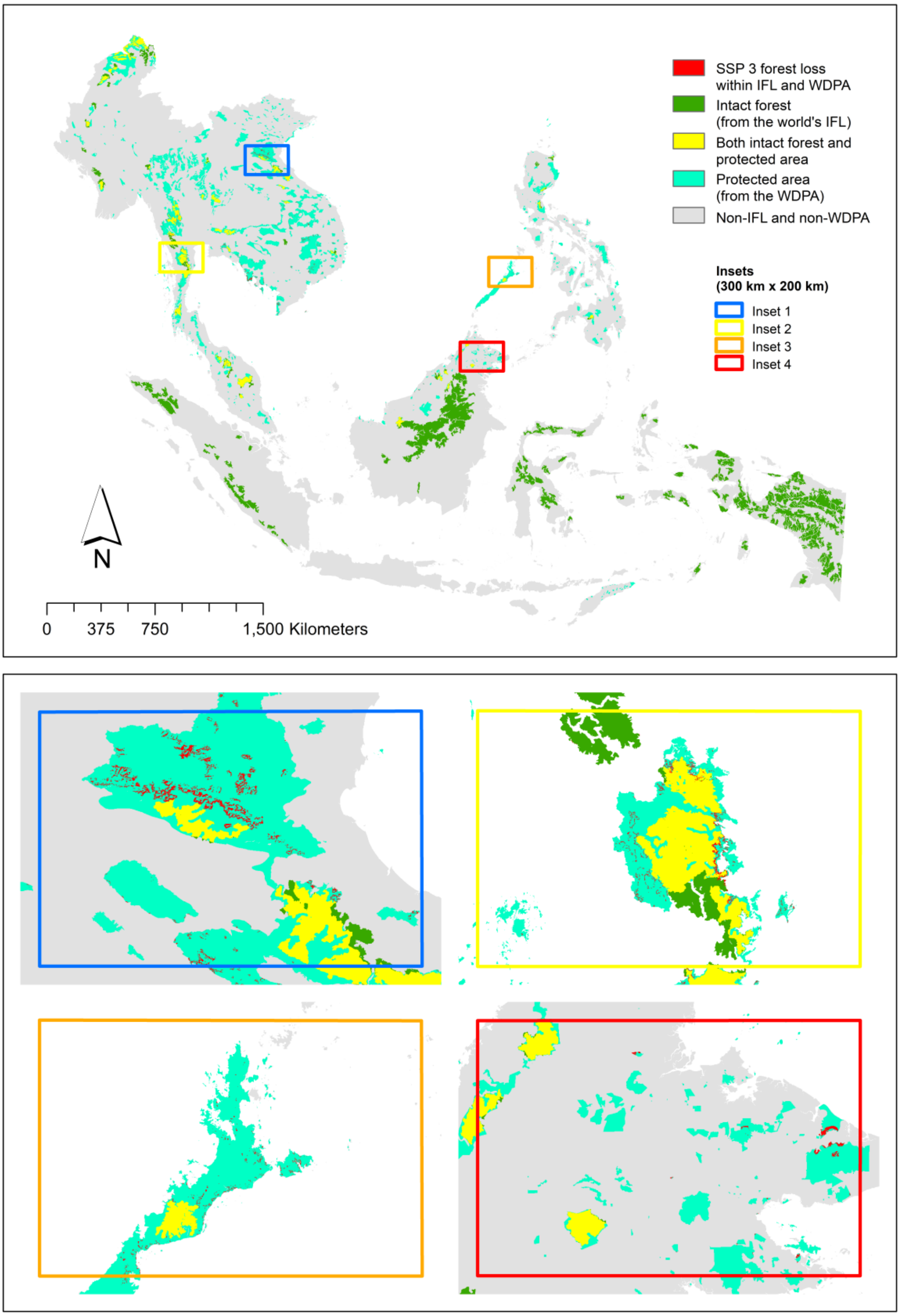

Supplementary Figure 4 Spatial distribution and extent of IFs and PAs in Southeast Asia (c. 2015). The maps also show the areas that are considered both IFs and PAs at the same time, as well as the projected forest cover losses under the worst-case scenario (SSP 3) (2015-2050). See Results and Supplementary Table 5 for corresponding statistics

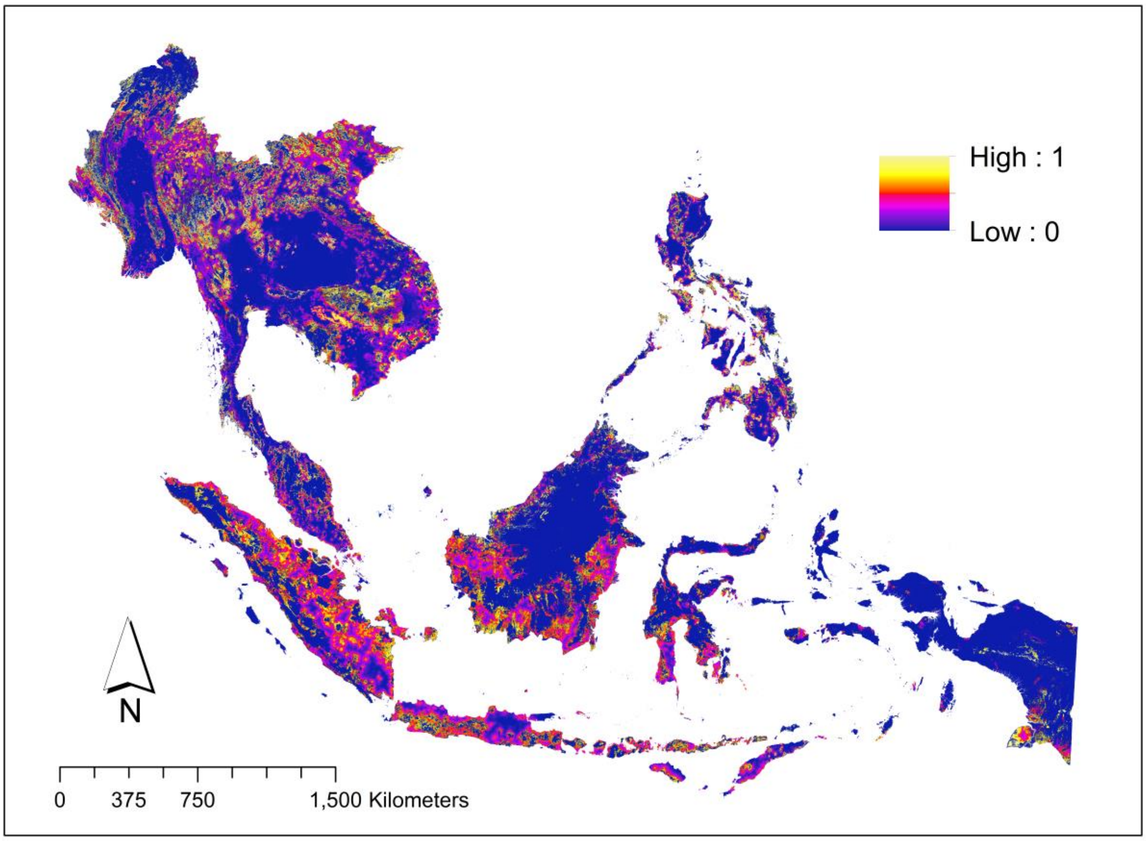

Supplementary Figure 5 Transition potential map (TPM) for forest cover gain (SSPs 1, 2 and 4). This is a mosaicked of the 11 country-level TPMs across the whole of Southeast Asia

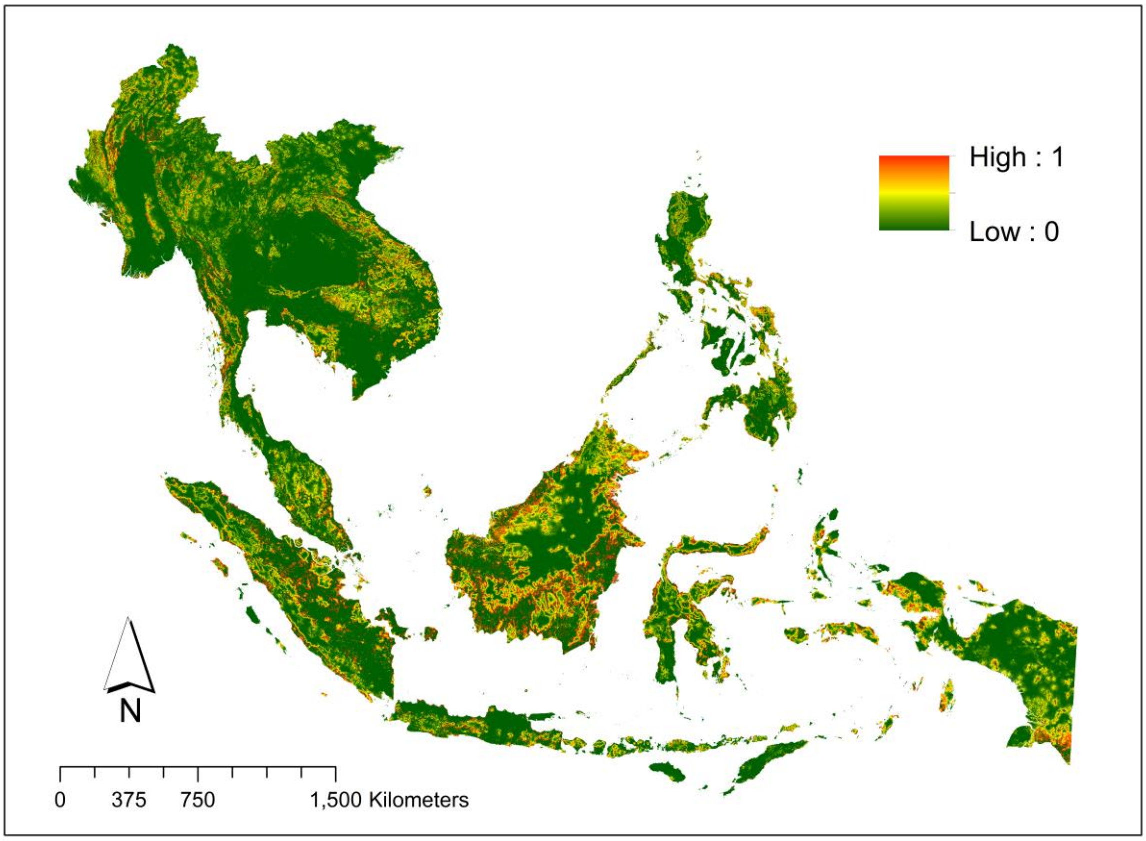

Supplementary Figure 6 Transition potential map (TPM) for forest cover loss (SSPs 3 and 5). This is a mosaicked of the 11 country-level TPMs across the whole of Southeast Asia

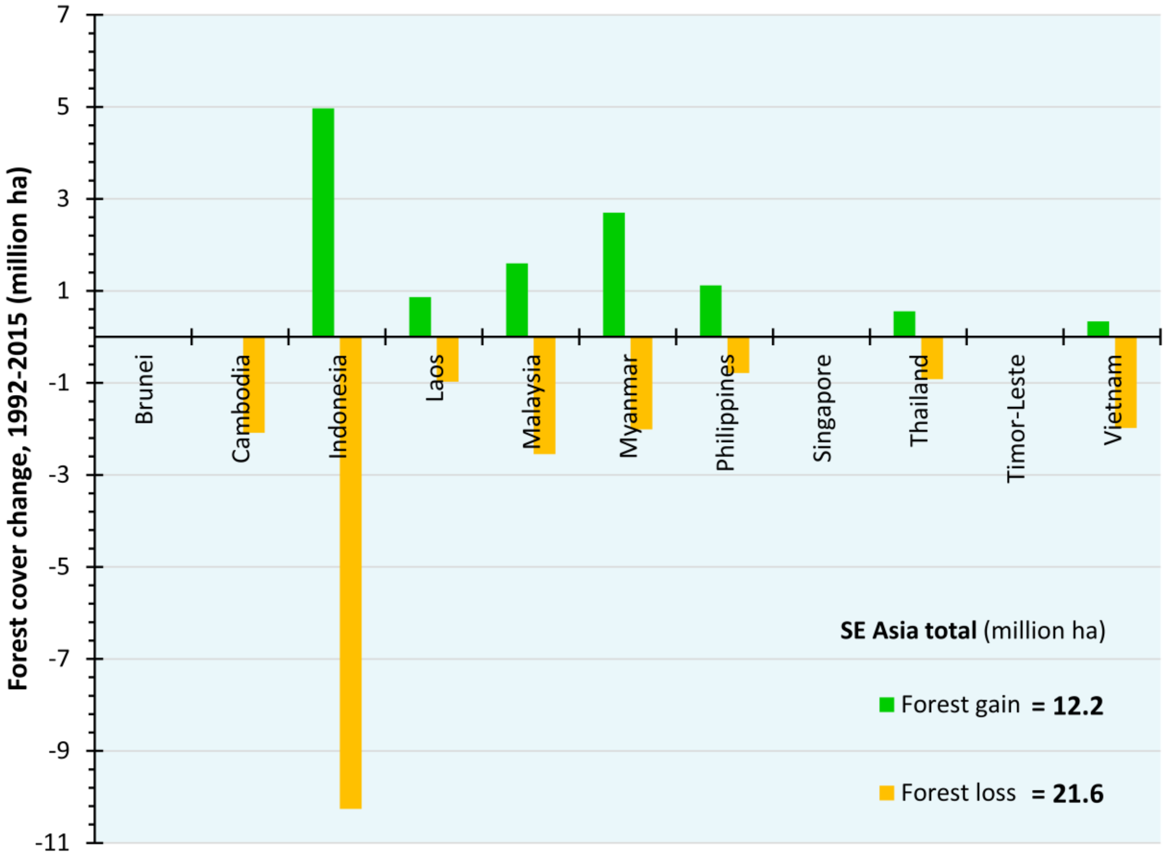

Supplementary Figure 7 Detected forest cover gains and losses in Southeast Asia (1992-2015). These statistics are based on the F/NF maps reclassified from the ESA-CCI land cover maps (see Methods)

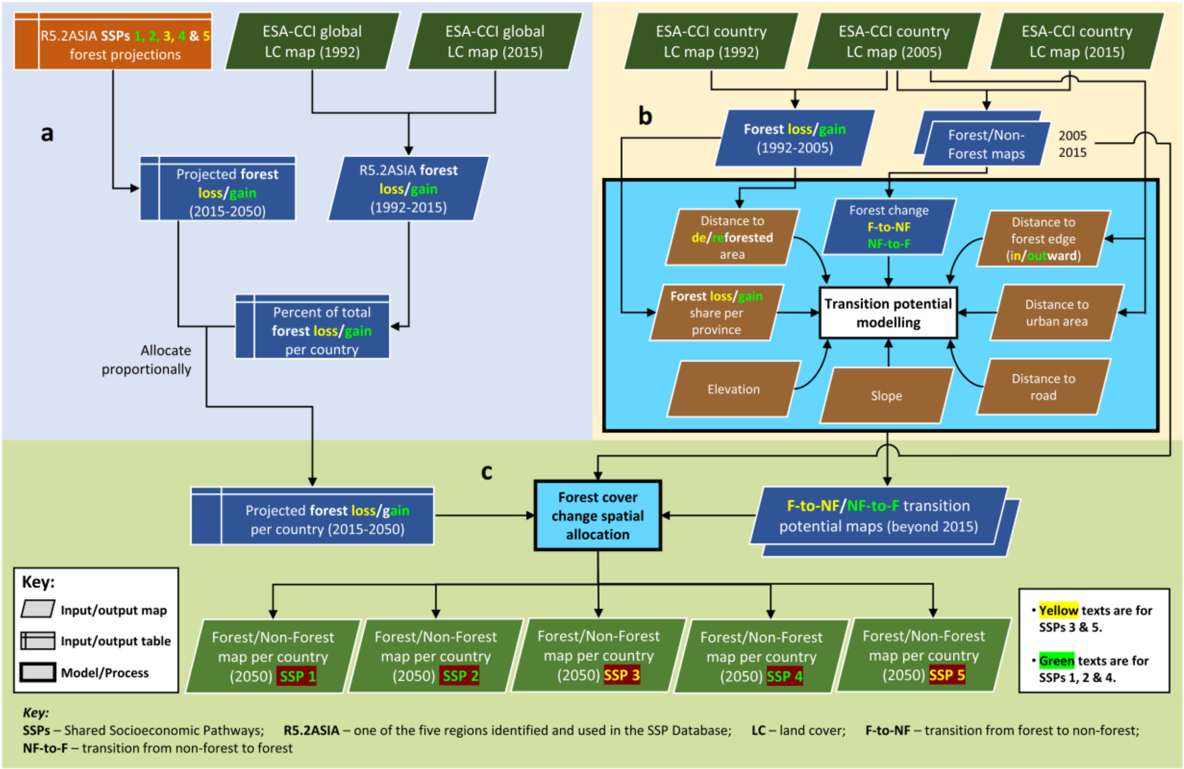

Supplementary Figure 8 Flowchart of the spatially explicit land change modelling procedure used to spatially allocate the projected quantities of forest cover changes under the five baseline SSPs. Forest cover change quantification (**a**), transition potential modelling (**b**) and forest cover change spatial allocation (**c**). The simulated 2050 forest/non-forest (F/NF) maps were spatially overlaid with the 2015 F/NF map to detect and extract the spatially allocated projected forest cover changes, i.e. gains for SSPs 1, 2 and 4, and losses for SSPs 3 and 5

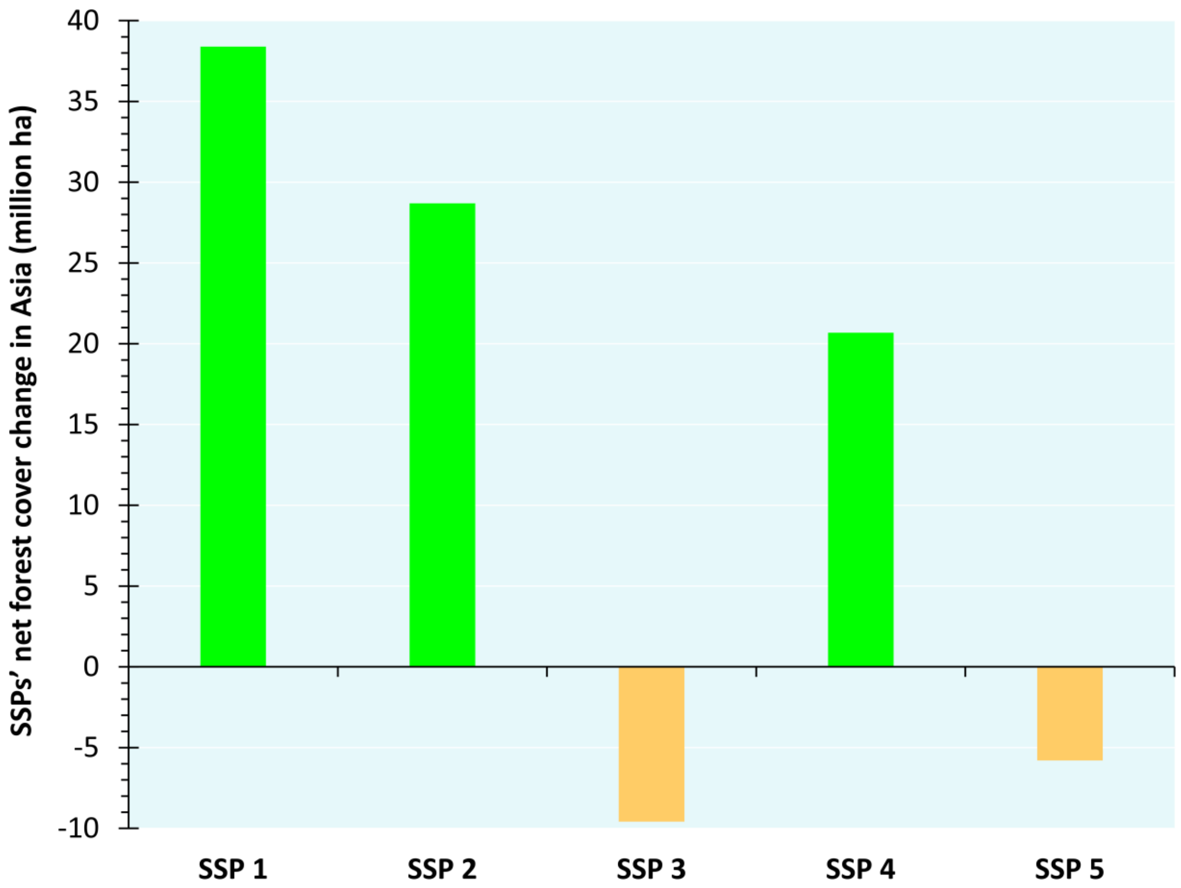

Supplementary Figure 9 Projected quantities of forest cover changes in Asia (one of the five SSP regions) under the five baseline SSPs (2015-2050). The 2015 projected forest cover refers to the average between the 2010 and 2020 projections. Data source: SSP Public Database, Version 1.1 (https://tntcat.iiasa.ac.at/SspDb)

Supplementary Table 1 Comparison of past-to-present (c. 2005-2015) gross forest and AFCS losses in Southeast Asia

	Forest Loss				AFCS Loss					
	[a] Based on ESA-CCI Land Cover Data (2005-2015)		Based on Hansen et al.'s Forest Loss Data (2005-2015)[1]		[a] Based on 2000 AGB and ESA-CCI Gross Forest Loss (2005-2015)		Based on 2000 AGB and Hansen et al.'s Gross Forest Loss (2005-2015)[1]		[b] Based on Baccini et al.'s Results (2004-2014)[2]	
**Country**	**M ha**	**%**	**M ha**	**%**	**Tg C**	**%**	**Tg C**	**%**	**Tg C**	**%**
Brunei	0.03	0.03	0.09	0.05	0.27	0.03	1.48	0.06	0.99	0.08
Cambodia	4.00	5.03	12.50	6.36	53.24	5.34	151.36	6.44	116.11	9.88
Indonesia	49.28	61.98	120.18	61.11	619.16	62.06	1440.90	61.35	556.21	47.34
Laos	3.00	3.77	7.10	3.61	38.25	3.83	80.77	3.44	85.93	7.31
Malaysia	13.18	16.57	37.03	18.83	173.51	17.39	470.94	20.05	184.11	15.67
Myanmar	4.18	5.26	7.46	3.80	46.01	4.61	80.55	3.43	113.28	9.64
Philippines	2.41	3.04	0.90	0.46	24.55	2.46	11.22	0.48	24.49	2.08
Singapore	0.01	0.01	0.00	0.00	0.04	0.00	0.02	0.00	No Data	No Data
Thailand	0.59	0.74	2.88	1.46	6.00	0.60	28.45	1.21	32.34	2.75
Timor-Leste	0.02	0.03	0.02	0.01	0.21	0.02	0.19	0.01	0.63	0.05
Vietnam	2.82	3.54	8.50	4.32	36.50	3.66	82.80	3.53	60.87	5.18
**SE Asia**	**79.51**		**196.67**		**997.73**		**2348.69**		**1174.96**	
**Annual**	**7.95**		**19.67**		**99.77**		**234.87**		**117.50**	

[a] These results are the ones discussed in the Results section: past-to-present forest and carbon stock losses. [b] These values are accumulated values over the 2004-2014 period derived from the Supplementary Table 1 of the cited source.

Supplementary Table 2 Country-level distribution of the projected forest cover and AFCS changes in Southeast Asia across the five baseline SSPs (2015-2050)

**Country**	**SSP 1**		**SSP 2**		**SSP 3**		**SSP 4**		**SSP 5**	
	Forest Gain (M ha)	AFCS Gain (Tg C)	Forest Gain (M ha)	AFCS Gain (Tg C)	Forest Loss (M ha)	AFCS Loss (Tg C)	Forest Gain (M ha)	AFCS Gain (Tg C)	Forest Loss (M ha)	AFCS Loss (Tg C)
Brunei	0.027	3.05	0.020	2.282	-0.002	-0.34	0.014	1.64	-0.001	-0.21
Cambodia	0.025	2.04	0.019	1.563	-0.499	-55.47	0.014	1.07	-0.302	-33.72
Indonesia	7.984	813.57	5.967	595.424	-2.457	-434.48	4.301	418.35	-1.487	-262.93
Laos	1.389	67.50	1.038	49.130	-0.234	-27.41	0.748	34.48	-0.141	-16.52
Malaysia	2.569	242.72	1.920	184.920	-0.610	-105.75	1.384	135.89	-0.369	-65.04
Myanmar	4.338	233.77	3.243	170.812	-0.482	-54.64	2.337	119.55	-0.291	-32.93
Philippines	1.799	192.28	1.344	143.386	-0.187	-31.95	0.969	102.94	-0.113	-19.36
Singapore	0.002	0.06	0.001	0.048	-0.001	-0.10	0.001	0.04	-0.001	-0.05
Thailand	0.894	56.30	0.668	41.547	-0.220	-23.58	0.481	29.74	-0.133	-14.17
Timor-Leste	0.040	3.38	0.030	2.535	-0.002	-0.16	0.022	1.87	-0.001	-0.08
Vietnam	0.545	36.30	0.408	27.117	-0.475	-56.13	0.294	19.57	-0.287	-33.66
**SE Asia**	**19.61**	**1650.96**	**14.66**	**1218.76**	**-5.17**	**-790.02**	**10.57**	**865.15**	**-3.13**	**-478.68**
**Annual**	**0.56**	**47.17**	**0.42**	**34.82**	**-0.15**	**-22.57**	**0.30**	**24.72**	**-0.09**	**-13.68**

Supplementary Table 3 Top 30 provinces in Southeast Asia in terms of the projected AFCS changes under the five baseline SSPs (2015-2050)

SSP 1			SSP 2			SSP 3			SSP 4			SSP 5		
Province	Country	AFCS Gain (Tg C)	Province	Country	AFCS Gain (Tg C)	Province	Country	AFCS Loss (Tg C)	Province	Country	AFCS Gain (Tg C)	Province	Country	AFCS Loss (Tg C)
Merauke	Indonesia	65.6	Merauke	Indonesia	59.1	Kutai Timur	Indonesia	20.0	Merauke	Indonesia	52.2	Kutai Kartanegara	Indonesia	12.1
Ketapang	Indonesia	35.8	Ketapang	Indonesia	24.6	Ketapang	Indonesia	19.2	Hkamti	Myanmar	18.8	Ketapang	Indonesia	11.5
Ogan Komering Ilir	Indonesia	31.2	Ogan Komering Ilir	Indonesia	23.8	Kutai Kartanegara	Indonesia	17.4	Ogan Komering Ilir	Indonesia	16.4	Bintulu	Malaysia	11.2
Hkamti	Myanmar	26.8	Hkamti	Myanmar	22.8	Bintulu	Malaysia	15.7	Ketapang	Indonesia	15.0	Kutai Timur	Indonesia	11.1
Myitkyina	Myanmar	25.1	Myitkyina	Myanmar	18.6	Kutai Barat	Indonesia	14.2	Seruyan	Indonesia	13.6	Selangau	Malaysia	8.8
Seruyan	Indonesia	21.7	Seruyan	Indonesia	17.6	Kotawaringin Timur	Indonesia	12.2	Myitkyina	Myanmar	13.1	Kutai Barat	Indonesia	8.8
Kutai Barat	Indonesia	15.5	Jayawijaya	Indonesia	12.3	Krâchéh	Cambodia	11.5	Jayawijaya	Indonesia	11.9	Krâchéh	Cambodia	8.3
Kawkareik	Myanmar	15.3	Mindat	Myanmar	12.0	Kaôh Kong	Cambodia	11.4	Boven Digoel	Indonesia	10.6	Kotawaringin Timur	Indonesia	7.7
Mindat	Myanmar	15.2	Beluran	Malaysia	11.9	Berau	Indonesia	10.6	Beluran	Malaysia	9.2	Kaôh Kong	Cambodia	7.6
Beluran	Malaysia	14.5	Sukamara	Indonesia	11.3	Barito Utara	Indonesia	10.5	Mindat	Myanmar	9.1	Nghệ An	Vietnam	7.5
Rokan Hilir	Indonesia	14.5	Kutai Barat	Indonesia	11.2	Selangau	Malaysia	10.5	Sukamara	Indonesia	8.5	Miri	Malaysia	7.5
Kotawaringin Barat	Indonesia	13.8	Kawkareik	Myanmar	11.2	Nghệ An	Vietnam	9.8	Quezon	Philippines	8.4	Berau	Indonesia	6.5
Quezon	Philippines	13.8	Quezon	Philippines	11.1	Rôtânôkiri	Cambodia	9.8	Marudi	Malaysia	8.2	Barito Utara	Indonesia	6.3
Sukamara	Indonesia	13.7	Boven Digoel	Indonesia	11.0	Miri	Malaysia	9.8	Kawkareik	Myanmar	7.9	Champasak	Laos	6.2
Mappi	Indonesia	13.6	Kotawaringin Barat	Indonesia	10.4	Pasir	Indonesia	9.7	Mappi	Indonesia	7.5	Rôtânôkiri	Cambodia	6.1
Kutai Kartanegara	Indonesia	12.7	Mappi	Indonesia	10.1	Pelalawan	Indonesia	9.3	Kutai Barat	Indonesia	7.2	Tanah Bumbu	Indonesia	6.1
Jayawijaya	Indonesia	12.5	Kutai Timur	Indonesia	9.6	Tanah Bumbu	Indonesia	9.3	Ma. Tenggara Barat	Indonesia	7.1	Pelalawan	Indonesia	6.0
Kutai Timur	Indonesia	12.4	Marudi	Malaysia	9.4	Kotawaringin Barat	Indonesia	9.1	Kutai Timur	Indonesia	7.0	Pasir	Indonesia	5.7
Musi Banyu Asin	Indonesia	11.7	Bintulu	Malaysia	8.7	Champasak	Laos	9.0	Bintulu	Malaysia	6.7	Kotawaringin Barat	Indonesia	5.6
Boven Digoel	Indonesia	11.4	Rokan Hilir	Indonesia	8.7	Bulongan	Indonesia	8.5	Gua Musang	Malaysia	6.7	Kawthoung	Myanmar	5.4
Bintulu	Malaysia	11.2	Kutai Kartanegara	Indonesia	8.5	Merauke	Indonesia	7.8	Kotawaringin Barat	Indonesia	6.4	Kampar	Indonesia	4.6
Marudi	Malaysia	10.8	Lamandau	Indonesia	8.2	Kampar	Indonesia	7.8	Tongod	Malaysia	6.3	Mergui	Myanmar	4.6
Banyuasin	Indonesia	10.8	Tongod	Malaysia	7.8	Kawthoung	Myanmar	7.4	Mukah	Malaysia	6.2	Merauke	Indonesia	4.5
Thandwe	Myanmar	10.0	Gua Musang	Malaysia	7.7	Quảng Nam	Vietnam	6.9	Lamandau	Indonesia	6.2	Bulongan	Indonesia	4.2
Lamandau	Indonesia	10.0	Ma. Tenggara Barat	Indonesia	7.7	Mergui	Myanmar	6.7	Melawi	Indonesia	6.1	Dawei	Myanmar	4.2
Loilen	Myanmar	9.9	Melawi	Indonesia	7.5	Katingan	Indonesia	6.4	Simunjan	Malaysia	5.7	Khammouan	Laos	4.0
Louang Namtha	Laos	9.5	Mukah	Malaysia	7.5	Khammouan	Laos	6.3	Louang Namtha	Laos	5.6	Kota Baru	Indonesia	4.0
Chiang Mai	Thailand	9.5	Louang Namtha	Laos	7.5	Kota Baru	Indonesia	6.1	Xaisômboun	Laos	5.5	Katingan	Indonesia	3.9
Kyaukme	Myanmar	9.5	Chiang Mai	Thailand	7.3	Dawei	Myanmar	5.9	Chiang Mai	Thailand	5.4	Quảng Nam	Vietnam	3.9
Tebo	Indonesia	9.4	Musi Banyu Asin	Indonesia	7.2	Pasaman Barat	Indonesia	5.4	Kutai Kartanegara	Indonesia	5.1	Pasaman Barat	Indonesia	3.9

Supplementary Table 4 Carbon (C) sequestration rates (CSRs) of forest classes and types across ecological zones and geographic locations in Southeast Asia (Mg C ha^{-1} yr^{-1})

Forest Classes and Types		Geographic locations		Remarks
		Continental	Insular	
**Old Growth Forest** [a]				
**Ecological zones**	Tropical rainforest	0.66	0.66	This 0.66 Mg C ha^{-1} yr^{-1} CSR for old growth forest is an average CSR derived from Qie et al.[3] (0.43 Mg C ha^{-1} yr^{-1} – based on long-term plot monitoring records from 1988 to 2010 in Borneo Island) and Pan et al.[4] (0.89 Mg C ha^{-1} yr^{-1} – the average between two estimates during the period of 1990-1999 and 2000-2007 for tropical Asia).
	Tropical moist forest			
	Tropical dry forest			
	Tropical shrubland			
	Tropical mountain system			
	Subtropical humid forest			
	Subtropical mountain system			
**Old Secondary Forest (> 20 years)**				
**Ecological zones**	Tropical rainforest	1.10	1.70	These CSRs are for tropical Asia taken from Table 4.9 of IPCC[5]. Values in **bold** refer to the middle values of value ranges.
	Tropical moist forest	1.00	1.50	
	Tropical dry forest	0.75	1.00	
	Tropical shrubland	0.65	0.50	
	Tropical mountain system	**0.38**	**1.00**	
	Subtropical humid forest	1.00	1.50	
	Subtropical mountain system	**0.38**	**1.00**	
**Young Secondary Forest (≤ 20 years)** [b]				
**Ecological zones**	Tropical rainforest	3.50	6.50	These CSRs are for tropical Asia taken from Table 4.9 of IPCC[5]. Values in **bold** refer to the middle values of value ranges.
	Tropical moist forest	4.50	5.50	
	Tropical dry forest	3.00	3.50	
	Tropical shrubland	2.50	1.00	
	Tropical mountain system	**1.50**	**3.75**	
	Subtropical humid forest	4.50	5.50	
	Subtropical mountain system	**1.50**	**3.75**	

[a] Only one CSR was used for old growth forest because more specific CSRs across ecological zones and geographic locations are lacking. [b] Each type of young secondary forest is further classified into annual sub-classes, from age 1 to age 20 (Supplementary Figure 2; Eq. (4)).

**Supplementary Table 5** Spatial extent of IFs and PAs (c. 2015) and the projected forest cover and AFCS losses (2015-2050)		
**(a) Spatial extent of IFs and PAs**		
	**Area (million ha)**	**Remarks**
IFs	38.3	18.6% (percentage relative to 2015 forest)
PAs	38.5	11.5% (percentage of 2015 forest inside PAs)
IFs within PAs	5.2	13.5% (percentage relative to total IFs)
**(b) Projected forest loss (thousand ha)**		
	**SSP 3**	**SSP 5**
IFs	39.2	22.3
PAs	580.0	362.0
**(c) Projected AFCS loss (Tg C)**		
	**SSP 3**	**SSP 5**
IFs	5.2	2.9
PAs	70.9	43.8

Supplementary Table 6 The Skill Measure (SM) values of the TPMs for forest gain and loss across the countries in Southeast Asia. See Methods and Results for interpretation

**Country**	**SSPs 1, 2 and 4**	**SSPs 3 and 5**
	**TPMs for forest gain**	**TPMs for forest loss**
Brunei	0.39	0.86
Cambodia	0.62	0.32
Indonesia	0.41	0.62
Laos	0.58	0.46
Malaysia	0.48	0.45
Myanmar	0.57	0.51
Philippines	0.59	0.41
Singapore	0.89	0.66
Thailand	0.77	0.50
Timor-Leste	0.79	0.46
Vietnam	0.54	0.41
**Average**	0.60	0.51
**Overall average = 0.56**		

Supplementary Table 7 Sample sizes used in transition potential modelling (unit: cell count at 300 m spatial resolution). One-half (50%) of the samples were used for training, while the other half (50%) were used for testing, in which the Skill Measure (SM) is based upon

**Country**	**SSPs 1, 2 and 4**	**SSPs 3 and 5**
	**TPMs for forest gain**	**TPMs for forest loss**
Brunei	**598**	**319**
Cambodia	**882**	10,000
Indonesia	10,000	10,000
Laos	**9379**	10,000
Malaysia	10,000	10,000
Myanmar	10,000	10,000
Philippines	10,000	10,000
Singapore	**65**	**137**
Thailand	10,000	**7285**
Timor-Leste	**480**	**291**
Vietnam	10,000	10,000

Note: Numbers in **bold** refer to the number of cells that transitioned from non-forest to forest (NF→F) (for SSPs 1, 2 and 4) and from forest to non-forest (F→NF) (for SSPs 3 and 5) from 2005 to 2015. Generally, it is not recommended to set the sample size higher than the smallest number of pixels that transitioned from one category to another as this leads to an unbalanced training procedure and the measured skill may be affected. In cases where the number of pixels that transitioned from one category to another is greater than 10,000 pixels, a sample size of 10,000 pixels is usually more than adequate, as per LCM MLP NN's documentation (TerrSet Help System[6]).

Supplementary Table 8 A two-by-two change matrix. Example: Indonesia

(a) For simulating forest cover gain (SSPs 1, 2 and 4)

Pixel count (300 m)

2015 \ 2050	Forest (F)	Non-Forest (NF)	Total 2015
Forest (F)	12,824,330	0	12,824,330
Non-Forest (NF)	887,096	7,285,294	8,172,390
Total 2050	13,711,426	7,285,294	

Proportion (input matrix)

2015 \ 2050	Forest (F)	Non-Forest (NF)	Total 2015
Forest (F)	1.0000	0.0000	1.0000
Non-Forest (NF)	0.1085[a]	0.8915[b]	1.0000
Total 2050	1.0000	1.0000	

[a] Derived by dividing NF2015-to-F2050 by the total NF2015 (also = 1 – 0.8915).

[b] Derived by dividing NF2015-to-NF2050 by the total NF2015 (also = 1 – 0.1085).

(b) For simulating forest cover loss (SSPs 3 and 5)

Pixel count (300 m)

2015 \ 2050	Forest (F)	Non-Forest (NF)	Total 2015
Forest (F)	12,551,300	273,030	12,824,330
Non-Forest (NF)	0	8,172,390	8,172,390
Total 2050	12,551,300	8,445,420	

Proportion (input matrix)

2015 \ 2050	Forest (F)	Non-Forest (NF)	Total 2015
Forest (F)	0.9787[c]	0.0213[d]	1.0000
Non-Forest (NF)	0.0000	1.0000	1.0000
Total 2050	1.0000	1.0000	

[c] Derived by dividing F2015-to-F2050 by the total F2015 (also = 1 – 0.0213).

[d] Derived by dividing F2015-to-NF2050 by the total F2015 (also = 1 – 0.9787).

References (Supplementary Information)

1 Hansen, M. C. et al. High-resolution global maps of 21st-century forest cover change. *Science* **342**, 850–853 (2013).

2 Baccini, A. et al. Tropical forests are a net carbon source based on aboveground measurements of gain and loss. *Science* **358**, 230–234 (2017).

3 Qie, L. et al. Long-term carbon sink in Borneo's forests halted by drought and vulnerable to edge effects. *Nat. Commun.* **8**, 1966 (2017).

4 Pan, Y. et al. A large and persistent carbon sink in the world's forests. *Science* **333**, 988–993 (2011).

5 IPCC (Intergovernmental Panel on Climate Change). *2006 IPCC Guidelines for National Greenhouse Gas Inventories. Vol. 4, Agriculture, Forestry and Other Land Use.* (Institute for Global Environmental Strategies (IGES), Japan, 2006).

6 TerrSet Help System. *TerrSet: Geospatial Monitoring and Modeling System.* (Clark Labs, Clark University, MA, USA, 2016).
