# Supplementary material for: The future of Southeast Asia’s forests
Source: Nat Commun. 2019 Apr 23;10:1829. doi: 10.1038/s41467-019-09646-4 (PMC6478739; doi:10.1038/s41467-019-09646-4)
Supplement: Supplementary file 3 — Reporting Summary [file 41467_2019_9646_MOESM3_ESM.pdf]

nature research

Corresponding author(s): DBPR NCOMMS-18-32527A

Last updated by author(s): Mar 20, 2019

Reporting Summary

Nature Research wishes to improve the reproducibility of the work that we publish. This form provides structure for consistency and transparency in reporting. For further information on Nature Research policies, see Authors & Referees and the Editorial Policy Checklist.

Statistics

For all statistical analyses, confirm that the following items are present in the figure legend, table legend, main text, or Methods section.

n/a	Confirmed	
☒	☐	The exact sample size (*n*) for each experimental group/condition, given as a discrete number and unit of measurement
☒	☐	A statement on whether measurements were taken from distinct samples or whether the same sample was measured repeatedly
☒	☐	The statistical test(s) used AND whether they are one- or two-sided<br>*Only common tests should be described solely by name; describe more complex techniques in the Methods section.*
☒	☐	A description of all covariates tested
☒	☐	A description of any assumptions or corrections, such as tests of normality and adjustment for multiple comparisons
☐	☒	A full description of the statistical parameters including central tendency (e.g. means) or other basic estimates (e.g. regression coefficient) AND variation (e.g. standard deviation) or associated estimates of uncertainty (e.g. confidence intervals)
☒	☐	For null hypothesis testing, the test statistic (e.g. *F*, *t*, *r*) with confidence intervals, effect sizes, degrees of freedom and *P* value noted<br>*Give P values as exact values whenever suitable.*
☒	☐	For Bayesian analysis, information on the choice of priors and Markov chain Monte Carlo settings
☒	☐	For hierarchical and complex designs, identification of the appropriate level for tests and full reporting of outcomes
☒	☐	Estimates of effect sizes (e.g. Cohen's *d*, Pearson's *r*), indicating how they were calculated

Our web collection on statistics for biologists contains articles on many of the points above.

Software and code

Policy information about availability of computer code

Data collection	No software was used.
Data analysis	ArcMap (ArcGIS) 10.5; TerrSet version 18.31; R version 3.2.5; Microsoft Excel 2016

For manuscripts utilizing custom algorithms or software that are central to the research but not yet described in published literature, software must be made available to editors/reviewers. We strongly encourage code deposition in a community repository (e.g. GitHub). See the Nature Research guidelines for submitting code & software for further information.

Data

Policy information about availability of data

All manuscripts must include a data availability statement. This statement should provide the following information, where applicable:

- Accession codes, unique identifiers, or web links for publicly available datasets
- A list of figures that have associated raw data
- A description of any restrictions on data availability

The sources of all the data used are given in the Methods section. All the geospatial data associated with the results presented, as well as the R code used, is available from the first and corresponding author upon request.

Field-specific reporting

Please select the one below that is the best fit for your research. If you are not sure, read the appropriate sections before making your selection.

☐ Life sciences ☐ Behavioural & social sciences ☒ Ecological, evolutionary & environmental sciences

October 2018

Ecological, evolutionary & environmental sciences study design

All studies must disclose on these points even when the disclosure is negative.

Study description	In this study, we focused on the spatial allocation of the projected quantities of future forest cover changes in Southeast Asia from 2015 to 2050 under the five baseline shared socioeconomic pathways (SSPs), employing a state-of-the-art spatially explicit land change modelling approach and using remotely sensed data. The potential implications of these spatially allocated projected forest cover changes were examined by quantifying their consequent aboveground forest carbon stock (AFCS) changes at the country and province levels, within the intact forests (IFs) and protected areas (PAs), and across forest classes in Southeast Asia.
Research sample	In this study, sampling was performed only during the transition potential modeling part. The generated sets of samples were used for training and testing (see Methods).
Sampling strategy	In relation to the above, a simple random sampling technique was employed by the Land Change Modeler (LCM), the land use model that we used which is available in TerrSet, a software package (see Methods). The generated sets of samples, including explanations, are given in Supplementary Table 7.
Data collection	The data used in this study were collected from various open sources, which have been acknowledged in the Methods section. The geospatial data were in raster and vector formats. The carbon sequestration rates were from a PDF (see IPCC 2006). The SSPs' projected forest quantities were from a Microsoft Excel file downloaded from the SSP Public Database, Version 1.1. All the data used were collected and managed by RCE, with some help from VA.
Timing and spatial scale	Based on the instructions given inside this box, this section is not applicable to our study.
Data exclusions	In the Methods section of our manuscript, we mentioned the availability of various land cover/forest cover and biomass datasets. However, we selected the ESA-CCI land cover datasets, including Hansen et al.'s forest loss dataset, and the 2010 Santoro et al. biomass dataset according to our data selection criteria and other explanations, which are both explicitly mentioned in the Methods section of our manuscript.
Reproducibility	To ensure the reproducibility of our study, we made sure that our Methods section is explicit and specific. We also produced two (2) flow diagrams detailing our approaches (Supplementary Figures 2 and 8).
Randomization	This is not applicable to our study. It is because we did not perform this type of analysis.
Blinding	This is not applicable to our study. It is because we did not perform this type of analysis.

Did the study involve field work? ☐ Yes ☒ No

Reporting for specific materials, systems and methods

We require information from authors about some types of materials, experimental systems and methods used in many studies. Here, indicate whether each material, system or method listed is relevant to your study. If you are not sure if a list item applies to your research, read the appropriate section before selecting a response.

Materials & experimental systems

n/a	Involved in the study	
☒	☐	Antibodies
☒	☐	Eukaryotic cell lines
☒	☐	Palaeontology
☒	☐	Animals and other organisms
☒	☐	Human research participants
☒	☐	Clinical data

Methods

n/a	Involved in the study	
☒	☐	ChIP-seq
☒	☐	Flow cytometry
☒	☐	MRI-based neuroimaging
